# Atomic Scale Analysis of the Enhanced Electro- and Photo-Catalytic Activity in High-Index Faceted Porous NiO Nanowires

**DOI:** 10.1038/srep08557

**Published:** 2015-02-24

**Authors:** Meng Shen, Ali Han, Xijun Wang, Yun Goo Ro, Alireza Kargar, Yue Lin, Hua Guo, Pingwu Du, Jun Jiang, Jingyu Zhang, Shadi A. Dayeh, Bin Xiang

**Affiliations:** 1 Department of Materials Science & Engineering, CAS key Lab of Materials for Energy Conversion, University of Science and Technology of China, Hefei, Anhui, 230026, China; 2 Department of Chemical Physics, University of Science and Technology of China, Hefei, Anhui, 230026, P. R. China; 3 Department of Electrical and Computer Engineering and Materials Science Program, University of California-San Diego, La Jolla, California 92093, USA; 4 Hefei National Laboratory for Physical Sciences at the Microscale, University of Science and Technology of China, Hefei, Anhui 230026, P. R. China; 5 National Center for Electron Microscopy, Lawrence Berkeley National Laboratory, Berkeley, California 94720, USA; 6 Molecular Foundry, Lawrence Berkeley National Laboratory, 1 Cyclotron Rd, Berkeley, CA 94720, USA; 7 Synergetic Innovation Center of Quantum Information & Quantum Physics, University of Science and Technology of China, Hefei, Anhui 230026, China

## Abstract

Catalysts play a significant role in clean renewable hydrogen fuel generation through water splitting reaction as the surface of most semiconductors proper for water splitting has poor performance for hydrogen gas evolution. The catalytic performance strongly depends on the atomic arrangement at the surface, which necessitates the correlation of the surface structure to the catalytic activity in well-controlled catalyst surfaces. Herein, we report a novel catalytic performance of simple-synthesized porous NiO nanowires (NWs) as catalyst/co-catalyst for the hydrogen evolution reaction (HER). The correlation of catalytic activity and atomic/surface structure is investigated by detailed high resolution transmission electron microscopy (HRTEM) exhibiting a strong dependence of NiO NW photo- and electrocatalytic HER performance on the density of exposed high-index-facet (HIF) atoms, which corroborates with theoretical calculations. Significantly, the optimized porous NiO NWs offer long-term electrocatalytic stability of over one day and 45 times higher photocatalytic hydrogen production compared to commercial NiO nanoparticles. Our results open new perspectives in the search for the development of structurally stable and chemically active semiconductor-based catalysts for cost-effective and efficient hydrogen fuel production at large scale.

Hydrogen production through water splitting is regarded as a promising approach for clean renewable hydrogen fuel generation, a promising pathway towards solving worldwide energy and environmental issues^1^,^2^,^3^,^4^. Semiconductor-based catalysts play an important role in the clean and cost-effective energy fuels due to their unique properties, which can be tailored by composition and surfaces for improved performance, and their abundance^5^,^6^,^7^. However, the efficiency of energy conversion in semiconductor catalysts is still low, which is mainly due to inefficient catalytic redox reactions^8^. Co-catalyst is utilized to reduce activation energy and improve the semiconductor-based catalyst activity. Noble metals (mainly Ag, Au, Pd and Pt) have traditionally been the popular candidates as effective co-catalysts^9^,^10^,^11^. However, the high cost of noble metals especially the most effective HER co-catalyst, Pt^12^, has largely hindered the commercialization progress. Recently metal-oxide co-catalysts such as Co_3_O_4_ and NiO^13^,^14^,^15^,^16^,^17^ have been actively studied. To achieve sufficient catalytic activity, extensive efforts have been made to increase the specific surface area by reducing the size of metal oxide nanoparticles^14^,^18^,^19^. But such an increase in surface area cannot on its own lead to the desired catalytic performance and other new or fundamental approaches are necessary.

The catalytic properties of metal oxides can be manipulated by modifying the surface structure^20^,^21^. For instance, the {110} surface of Co_3_O_4_ was found to be much more catalytically active than the {100} surface^20^. Generally, compared to the low index facets (LIFs), the high index facets (HIFs) have higher surface energies which are therefore not energetically favored to appear at surfaces in equilibrium^11^. However, the presence of surface defects (steps) can reduce the surface energy, leading to the concurrent occurrence of stable HIFs at curved surfaces^22^. It becomes crucial to quantitatively evaluate the role of those surface defects and the enhancement of HIF in catalytic performance. In this work, we report a quantitative correlation of the catalytic performance and density of exposed HIF atoms extracted from well-controlled experiments. The atomic structure investigations of the porous NiO nanowires (NWs) were carried out by spherical-aberration-corrected HRTEM. NiO catalytic performance as a function of exposed HIF atom density was confirmed by conducting electro and photocatalytic HER experiments.

The porous NiO NWs were synthesized by an electrospinning method (Figure S1 and Supporting Information for details). The morphology and microstructure of the NiO NWs were investigated as shown in Figure 1a–c. The NiO product maintains 1D shape of a porous architecture with a diameter of ~270 nm, composed of interconnected nanocrystals and numerous pores. The HRTEM technique enables us to further investigate the NiO NW atomic structure revealing the curved surfaces with many zig-zag features that emerged on the NiO nanocrystal surfaces (Figure 1d and e).

To probe the mechanism of the porous NiO NW formation, a thermogravimetry analysis (TGA) was carried out to investigate the pyrolysis of the as-electrospun Ni(CH_3_COO)_2_/PVP composite NWs at a heating rate of 10°C/min in air (Figure S2a, Supporting Information). The initial rapid weight decrease can be assigned to solvent evaporation at the stabilization stage (step I). A pre-oxidation stage (Step II) comprised a dehydration process of Ni(CH_3_COO)_2_ as a result of solid phase formation (1 − x)Ni(CH_3_COO)_2_ · xNi(OH)_2_. However, it only occurred in a surface-acetate hydrolysis, which led to moderate weight loss with a residual weight of ~80% in the temperature interval of 150°C−280°C (Figure S2a, Supporting Information). At a calcination stage (Step III), a major thermal decomposition happened in the composite NWs with a weight loss of 80%. The differential thermogravimetry (DTG) curve (Figure S2a, Supporting Information) suggests that the major decomposition was initiated at ~320°C. We attribute the sharp decrease in weight to the completed degradation of PVP and entire decomposition of (1 − x)Ni(CH_3_COO)_2_ · xNi(OH)_2_. The residual weight was a result of the formation of NiO NWs (20% in weight). To further study the mechanism of the NiO NW formation, X-ray photoelectron spectroscopy (XPS) analysis was conducted on the samples prepared at different temperatures (Figure S2b–d, Supporting Information), in which the evolution of the observed elemental peaks in XPS confirmed that there was only NiO phase existing in the calcination product. In addition, there was no distinguishable absorption bands of OH, CH_3_, C = O, C-N, and C-O groups observed in the Fourier–transform infrared spectroscopy (FTIR) of the calcinated NWs (Figure S3, Supporting Information). Therefore, these combined results corroborate that the formation of the porous NiO NWs is based on the volume loss and gas release.

In equilibrium, the shape of a crystal always tends to minimize the total surface energy. In terms of the facet stability, the higher the surface energy of the facets, the less stable the facets are and this is frequently observed in crystal growth where growth rates in the direction perpendicular to high surface energy plane are usually much faster than other planes^11^. Therefore, it is not favorable to achieve high-index facets on the surfaces during the NiO crystal growth, resulting from the meta-stable interface caused by high surface energy. However, involving lattice defects (such as steps, impurities et al.) is an effective strategy to reduce the energy barrier for nucleation of new NiO crystal layers and thus constitutes the active sites. The presence of surface defects gives rise to a surface strain field^9^, that leads to the occurrence of the stable high surface energy facets. In NiO NW synthesis described above, a non-equilibrium calcination process was employed to prepare the porous NiO NWs. At the calcination stage, the thermal decomposition effect on Ni(CH_3_COO)_2_/PVP composite triggers the nucleation and growth of NiO. In the meanwhile, small voids are generated at the interface due to the gas phase desorption from the oxidation reactions between the inner carbon, nitrogen, hydrogen atoms and atmospheric oxygen. Numerous vacancies tend to modify the interfacial tension, which exerts a profound impact on the development of the NiO surface structures^9^. As a result, NiO nanocrystals form into stepped surfaces composed of high density of defects. In the presence of high density of surface defects, the high surface energy facets could prevail at equilibrium state. The non-equilibrium calcination process plays an important role in the formation of surface steps. We use "NiO_10%", "NiO_30%" and "NiO_50%" notations in reference to porous NiO NWs synthesized using 10 wt%, 30 wt% and 50 wt% nickel acetate tetrahydrate (Ni(CH_3_COO)_2_ · 4H_2_O) (see details in Supporting Information and Table S1). To probe the global features of the NiO, we conducted a series of TEM experiments to analyze the nanoparticle surface along different orientations. We carried out the experiments in a JEM-ARM 200F JEOL TEM which has tilt angles a and b limited to the range of −22° to +22°, respectively. This should in principle allow us to rotate the sample along the [120] and [130] zone axis. However, during our TEM experiments, we found the exposed nanoparticles in the nanowires are mostly along [110] zone axis within a small tilted angle (±5°). As a result, only [120] direction becomes the other available zone axis. Therefore, our TEM experiments presented in Figure 2 were carried out in two zone axes, the [110] and the [120]. Fig. 2 reveals that 30%_NiO has the highest density of surface defects with a zone axis along [110] direction (Figure S4). Along the [120] direction, we are only able to achieve one-dimensional lattice fringes instead of two-dimensional atomic scale images (Figure 2e–h). The atomic plane distance of the high-index (420) planes is 0.9Å, much smaller than the 2.1Å atomic plane distance of the (002) lattice plane. It is therefore more challenging to resolve the (420) high index lattice fringes in TEM compared to (002) plane. Therefore we can only observe the (002) lattice fringes of instead of two-dimensional atomic scale images in the [120] zone orientation which precluded counting the exposed high-index atoms on each nanoparticle surfaces along the [120] zone axis from Figure 2e–h. However, we can still qualitatively analyze that higher density of surface defects are exposed in 30%_NiO nanoparticles along the [120] zone axis.

Surface area HRTEM studies in Figure 3 demonstrate that the NiO nanocrystal surfaces are composed of ordered terraces separated by monatomic or multi-atomic height steps. The indexing of the surface structures indicates that NiO nanocrystal surfaces are terminated by the HIFs and LIFs (Figure 3a–d, notation details in Supporting Information). As discussed above, the exposed HIFs can be stable in the presence of surface defects such as steps. The larger width of low-index terrace generally results in an exposed higher index facet^23^. While the HRTEM provides a 2D projection of the NiO nanoparticles, we hypothesize that the terrace steps extend uniformly in the third dimension as pictured in the cartoon models in Figure 3e–g. We define the linear density of exposed HIF atoms of NiO nanocrystal as the number of exposed HIF atoms divided by the projected plane perimeter. Our calculation (Figure S5, Supporting Information) indicates that NiO_30% has the highest density of exposed HIF atoms with a value of 2.92 × 10^7^/cm while the commercial NiO has the lowest density with a value of 1.24 × 10^6^/cm. NiO_10% and NiO_50% have a density of exposed HIF atoms with 1.25 × 10^7^/cm and 8.32 × 10^6^/cm, respectively. The surface structures of as-synthesized porous NiO NW were extensively modified by the defects, compared to commercial NiO. The volume loss and gas release processes could be responsible for the difference in the density of exposed HIF atoms in NiO_10%, NiO_30% and NiO_50% samples. Figure 3h shows the calculated energy difference for electron transfer from NiO to reduce H^+^ to H_2_ in water, 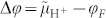, where 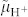 is the electrochemical potential of H^+^ in water with respect to the vacuum level (4.085 eV) and *φ_F_* is the work function at the high index facet (obtained from first principles using density functional theory, Supporting Information). The lower *Δφ* indicates a lower energy barrier for electrons to transfer from the NiO to reduce H^+^ to H_2_ in water, and the minima in *Δφ* are exhibited for the NiO_30%.

Fundamentally, the difference in work function between HIF and adjacent low index terrace results in modulation of the spatial charge distribution of the neighboring surface facets^24^,^25^,^26^, and induces an electric field between the HIF and adjacent low index terrace. In the presence of the induced electric field, the decomposition of the adsorbed polarized molecules can be enhanced in the HER. In addition, the exposed HIF unique local structure environment also provides active sites for breaking chemical bonds^23^. Generally, the potential for a surface ion *i* contributing to surface potential of an ionic crystal can be written by^27^:
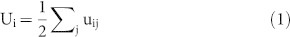
where u_ij_ is the potential induced by interaction between ion *i* and surrounding *j* ions. u_ij_ can be expressed as: 
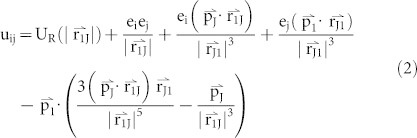
where r_ij_ is the displacement vector from ion j to i, e_i_, e_j_ represent the ionic charges, p_i_, p_j_ are the induced electric dipole moments. 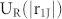 stands for the short-range repulsive potential only for nearest neighbors. The second term is the long-range Coulomb potential. The last three terms represent the polarization energy due to the presence of surface induced dipoles. The surface polarization electric field E_Di_ on ion i can be written as
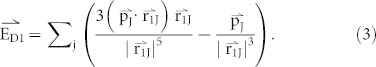
Falicov *et al.* have reported that only ions on the surface have non-zero dipoles while ions in bulk have zero dipoles^25^. Nickel oxide has a face-centered cubic (FCC) structure with octahedral Ni^2+^ and O^2−^ sites, which is an ionic crystal. In a unit cell, each Ni^2+^ has six nearest-neighbor ions. However, in terms of surface atoms, the Ni^2+^ ion has a lower number of closest-neighbors due to the non-periodicity in the surface structure. In addition, HIF has lower symmetry compared to LIF^23^. Generally, for ionic solids, HIFs have high concentration of low coordination number sites (steps and kinks) while LIFs have low concentration of low coordination number sites^28^. With the presence of high index facets on the surface, the low symmetries and distortions of the surface structure induce non-zero dipoles on the terrace of the stepped surface. Higher density of exposed HIF atoms provides more non-zero dipoles at the step and kink sites, leading to larger electric dipole moments. As a result, a stronger polarization electric field is induced at the surface, which is simply the summation of each surface dipole field over all ions on the surface as indicated in Eq. (3). A large local electric field on the surface assists in polarizing the incoming molecules with well-defined polarizability. Subsequently the covalent bonds in incoming molecules are broken apart, thereby facilitating chemical reactions. Additionally, a high density of surface defects (steps, kinks) favors the collisions between the reaction molecules and surfaces. The reaction probability can thus be increased by several orders of magnitude^28^.

To evaluate the catalytic performance of as-synthesized NiO NWs, we first utilized the porous NiO NWs as catalyst for the electrocatalytic HER. Figure 4a shows the electrocatalytic activity of porous NiO NWs with different densities of exposed HIF atoms by the same mass loading of 0.28 mg/cm^2^ onto the fluorine-doped tin oxide (FTO) substrates. The electrochemical catalytic performance follows an order of NiO_30% > NiO_10% > NiO_50% > commercial NiO. Tafel plot (Figure S7) indicates that NiO_30% sample has a small slope despite there is a large overpotential of ~800 mV. Since the morphology of the as-synthesized NiO is porous NWs, there is probably some pore blockage by evolved hydrogen gas and effective reduction in the electrode active surface area which might lead to the observed large overpotential^29^. Significantly, the Faradaic efficiency curve of NiO_30% measured under a fixed potential of −0.88 V vs. RHE (Figure 4b) shows that the amount of hydrogen evolved is in accordance with the amount of hydrogen expected on the basis of 100% Faradaic efficiency, implying a high efficiency of charge transfer that facilitates the HER.

The stability of catalyst is another important requirement. The stability of the electrochemical activity of NiO_30% was measured by chronopotentiometry under a fixed current density of 10 mA/cm^2^ (inset of Figure 4b). No significant change of the overpotential during the catalytic performance was observed. To further probe the correlation of surface structure and catalytic activity, we utilized as-synthesized NiO NWs with different densities of exposed HIF atoms as co-catalyst for the photocatalytic HER. Figure 4c shows different hydrogen production yield achieved in the photocatalytic HER, in which NiO_30% produced 45 times more hydrogen yield compared to the commercial NiO. In the absence of the as-synthesized NiO, the hydrogen production exhibits a substantial decrease (Figure S8, Supporting Information). The stable performance of NiO_30% as co-catalyst (Figure 4c inset) shows the amount of hydrogen gas is linearly dependent on time even after 12 hours of light irradiation, in which there was no significant rate decrease. Figure 4d shows a profile of hydrogen production as a function of NiO_30% weight percentage undergoing visible light irradiation (λ > 420 nm). The amount of hydrogen production rises with a weight increase in NiO_30% and it reaches the highest value of 90 μmol with amount of 2 wt% NiO_30%. Further increase in the amount of NiO_30% resulted in a significant decrease in the hydrogen production yield. An excess weight percentage of NiO_30% could screen the active sites exposed on the surface and could also block the visible light absorbing material, leading to relatively poor photocatalytic performance. This non-linear behavior between the catalytic efficiency and the ratio of catalyst loading was also observed in other semiconductor-based photocatalysts loaded by different co-catalysts^30^. The inset HRTEM image demonstrates the stability of NiO_30% as co-catalyst after the photocatalytic HER where the highly exposed surface steps in the NiO_30% sample prevail. This significant stability test validates for the first time, that those high-index facets responsible for the enhanced HER are conserved after long durations of reaction time. In addition, the crystal structure evolution of the dye-sensitized TiO_2_ materials loaded with NiO_30% was also monitored by X-ray diffraction (XRD) (Figure S10, Supporting Information). The indexed XRD patterns confirm that the crystal phases of the NiO and TiO_2_ coexist all the time before and after hydrogen production experiments. It suggests excellent stability of porous NiO for the photocatalytic HER.

Figure 5 summarizes the photo- and electrocatalytic HER performance as a function of exposed HIF atom density. The measured BET value (Table S2, Supporting Information) is within the the same order of magnitude in different NiO nanowires with slightly higher specific areas for the 10% and 50% NiOx nanoparticles compared to the 30%. This suggests that the specific surface area has lower or negligible influence on the catalytic activity in our NiO nanowires compared to surface defects. Under the effects of the induced electric fields at high-index facets, the decomposition of the adsorbed polar molecules is markedly enhanced, resulting in the increased chemical activity. In addition, the adsorbed molecules have a larger number of nearest neighbor ions at a step site compared to a flat surface^31^. Therefore, there is an increased availability at the step site for adsorption and reaction. In terms of activation energy, the active sites reduce the potential energy in the chemical reaction by forming temporary chemical bonds with the adsorbed molecules. Providing more active sites, larger density of exposed HIF atoms induces a stronger polarization electric field arising from the summation of all adjacent surfaces and leads to a substantial enhancement of catalytic performance in both photocatalytic and electrocatalytic HERs.

In summary, porous NiO NWs with highly exposed HIF were successfully synthesized via a cost-effective electrospinning method. Atomic-scale HRTEM analysis revealed that the high density of surface atomic steps conduced the exposure of the high-energy surfaces. The as-synthesized 1D porous NiO NWs exhibited high performance for both electrocatalytic and photocatalytic HER. The obtained correlation of exposed HIF density and catalytic performance provides guidance to engineer the surface structure to maximize the catalytic activity in the future for different material systems. Our study shows a potential route for the development of structurally stable and chemically active catalysts in new energy applications with high performance.

## Author Contributions

B.X. initiated the study and designed the experiments. M.S., A.H., Y.L. performed the experiments. X.W., J.J. carried on theoretical calculations. B.X., J.Z., J.J., Y.R., A.K., H.G., P.D. and S.A.D. analyzed the data. B.X., J.Z., P.D., S.A.D. prepared the manuscript. All the authors contributed to discussions of the project and writing of the manuscript.

## Supplementary Material

Supplementary Information Supporting Information

## Figures and Tables

**Figure 1:**
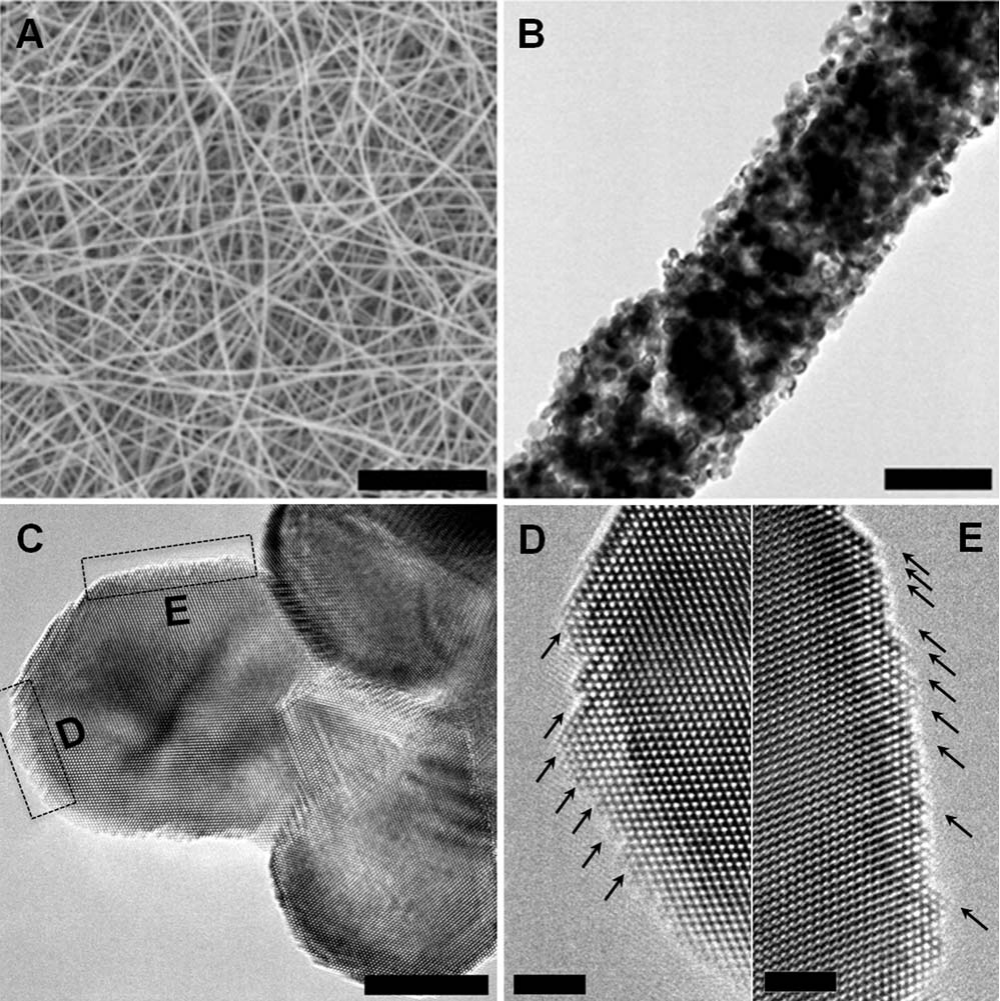
The morphology and microstructure of as-synthesized NiO NWs. SEM (a) and TEM (b) images of the as-synthesized NiO NWs. (c) HRTEM image of the as-synthesized NiO NWs composed of nanocrystals. Scale bar is 10 nm. (d) and (e) Atomic-scale surface structure analysis of the as-synthesized NiO NW. The observed curved surfaces consist of terraces, atomic steps. Scale bar is 2 nm.

**Figure 2:**
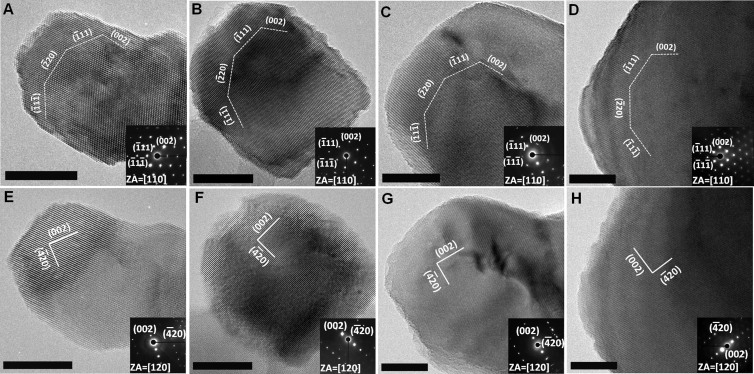
Surface structure analysis of the same NiO nanoparticles imaged at two different zone axes. Representative HRTEM images of a) NiO_10%, b) NiO_30%, c) NiO_50% and d) commercial NiO with the zone axis of [110] direction. e) NiO_10%, f) NiO_30%, g) NiO_50% and h) commercial NiO with the zone axis of [120] direction. Scale bar is 10 nm.

**Figure 3:**
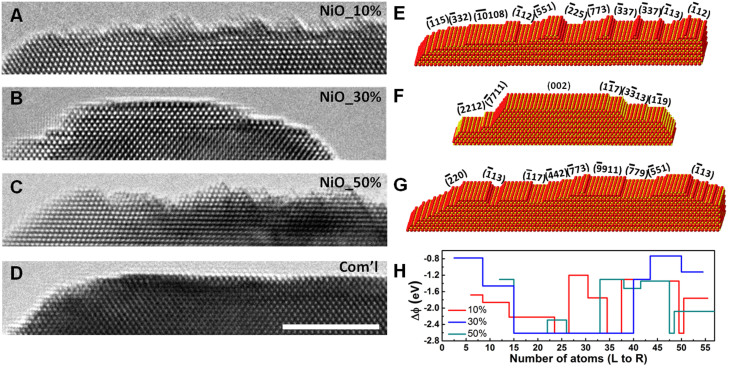
HRTEM images detailing surface structure of (a) NiO_10% with linear density of exposed HIF atoms of 1.25 × 10^7^/cm, (b) NiO_30% with linear density of exposed HIF atoms of 2.92 × 10^7^/cm, (c) NiO_50% with linear density of exposed HIF atoms of 8.32 × 10^6^/cm, (d) commercial NiO with linear density of exposed HIF atoms of 1.24 × 10^6^/cm. Scale bar is 5 nm. 3D models for the structure of the NiO nanoparticles for NiO_10% (e), NiO_30% (f) and NiO_50% (g). The step facets have been clearly indexed (detailed notation in Supporting Information). (h) The calculated energy difference between NiO high-index nanofacets and the electrochemical potential for H^+^ in water for NiO_10%, NiO_30% and NiO_50%. The x-axis represents the number of atoms starting at the left most side of the cartoons. The lower energy difference facilitates electron transfer from the NiO to water for hydrogen reduction as is predominant for the NiO_30% sample.

**Figure 4:**
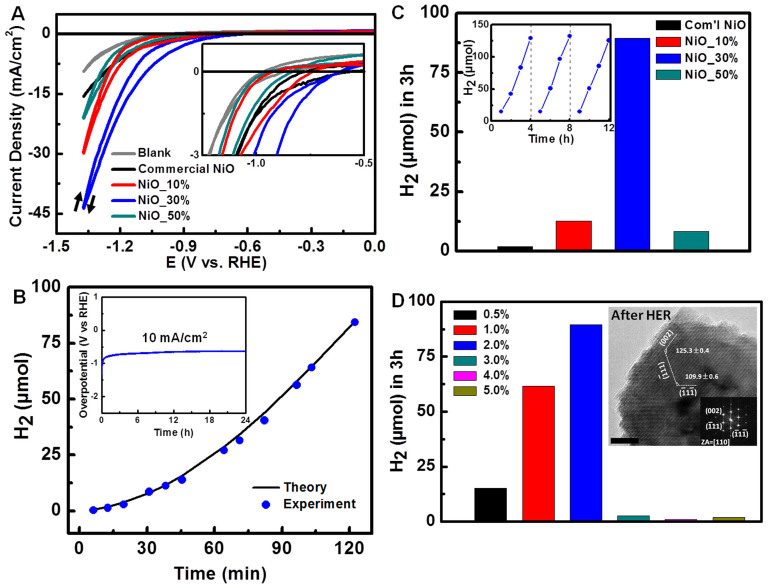
(a) The electrocatalytic activity of as-synthesized NiO NWs with different density of exposed HIF atoms. Inset is the zoom-in area. (b) The amount of hydrogen evolved by NiO_30% over time based on the performed experiment and calculation. Inset shows the electrocatalytic stability performance of the NiO_30% sample at a constant current density of 10 mA/cm^2^. (c) The amount of hydrogen evolution obtained by employing different density of exposed HIF atoms in the photocatalytic HER. Inset exhibits the stability performance of NiO_30% as co-catalyst in the photocatalytic HER. (d) The amount of hydrogen evolution as a function of NiO_30% loading mass ratio. The inset is the HRTEM image of NiO_30% sample after the photocatalytic HER demonstrating stability of the high-index facets. Scale bar of inset is 5 nm.

**Figure 5:**
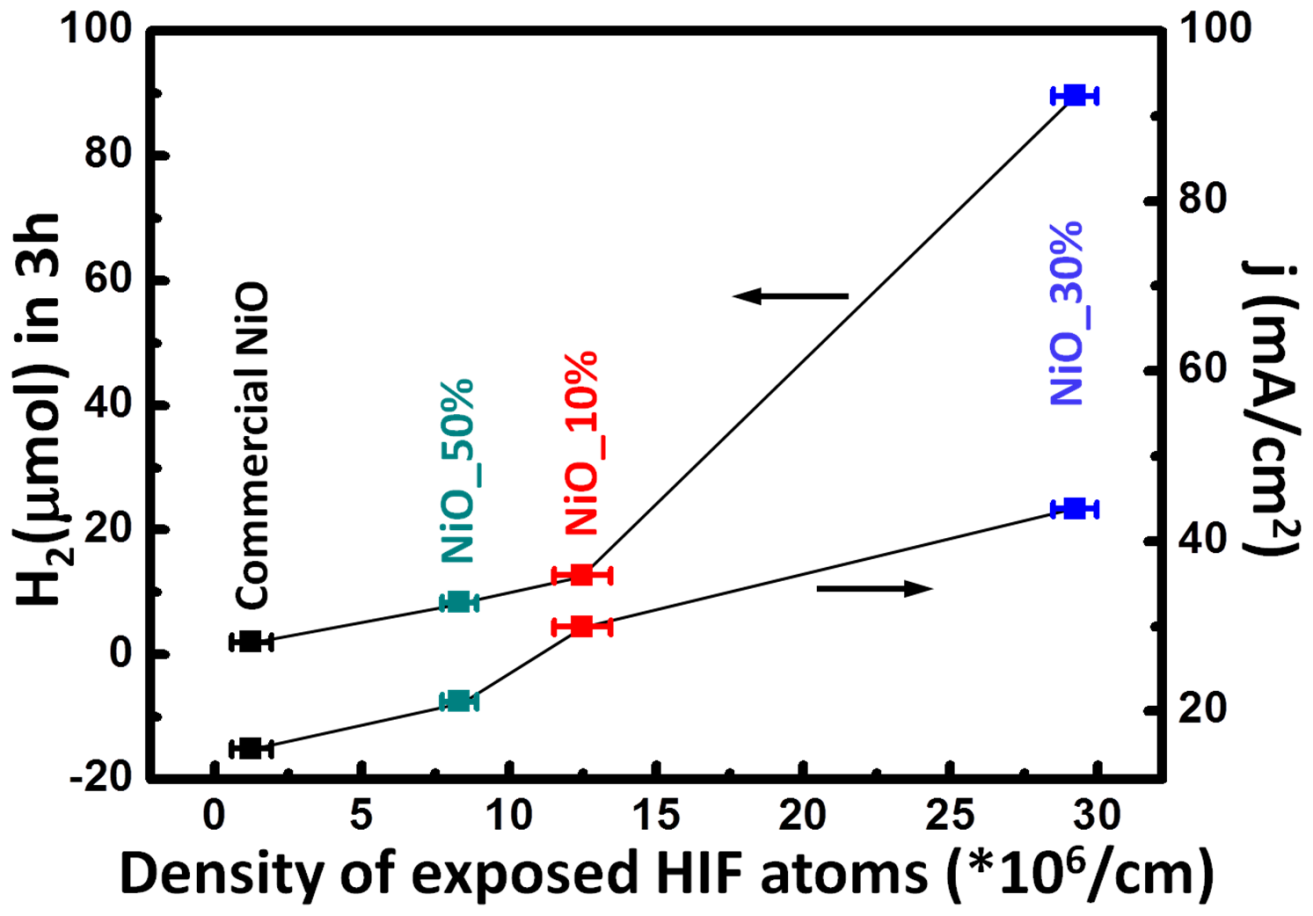
The quantitative relationship between catalytic performance and linear density of exposed HIF atoms in both photocatalytic and electrocatalytic HERs. The NiO catalytic performance shows a strong dependence on the density of exposed HIF atoms.
