# Supplementary material for: Atomic Scale Analysis of the Enhanced Electro- and Photo-Catalytic Activity in High-Index Faceted Porous NiO Nanowires
Source: Sci Rep. 2015 Feb 24;5:8557. doi: 10.1038/srep08557 (PMC4338422; doi:10.1038/srep08557)
Supplement: Supplementary Information — Supporting Information [file srep08557-s1.pdf]

Atomic Scale Analysis of the Enhanced Electro- and Photo-Catalytic Activity in High-Index Faceted Porous NiO Nanowires

Meng Shen,[1#] Ali Han,[1#] Xijun Wang,[2#] Yun Goo Ro,[3] Alireza Kargar,[3] Yue Lin,[4] Hua Guo,[5] Pingwu Du,[1*] Jun Jiang,[2] Jingyu Zhang,[6] Shadi A. Dayeh[3] and Bin Xiang[1,7*]

[1]Department of Materials Science & Engineering, CAS key Lab of Materials for Energy Conversion, University of Science and Technology of China, Hefei, Anhui, 230026, China
[2]Department of Chemical Physics, University of Science and Technology of China, Hefei, Anhui, 230026, P. R. China
[3]Department of Electrical and Computer Engineering, University of California San Diego, La Jolla, California 92093, USA
[4]Hefei National Laboratory for Physical Sciences at the Microscale, University of Science and Technology of China, Hefei, Anhui 230026, P. R. China
[5]National Center for Electron Microscopy, Lawrence Berkeley National Laboratory, Berkeley, California 94720, USA
[6]Molecular Foundry, Lawrence Berkeley National Laboratory, 1 Cyclotron Rd, Berkeley, CA 94720, USA
[7]Synergetic Innovation Center of Quantum Information & Quantum Physics, University of Science and Technology of China, Hefei, Anhui 230026, China

[#]These authors contributed equally to this work.
[*]Corresponding authors: binxiang@ustc.edu.cn, dupingwu@ustc.edu.cn

Porous NiO nanowires synthesis

Nickel acetate tetrahydrate ($Ni(CH_3COO)_2 \cdot 4H_2O$) was dissolved in N,N-Dimethylformamide (DMF) and was followed by the addition of 12.5wt% polyvinylpyrrolidone (PVP) to the solution which was stirred for 12 hours to get a green homogeneous solution. A high voltage power supply was used to provide a 15 kV high voltage for the as-prepared electrospinning solution. The feeding rate and the distance between the needle and the collector was 0.5 ml/h and 20 cm, respectively. The prepared nanowires were collected on an aluminum foil collector. The as-prepared PVP/($Ni(CH_3COO)_2 \cdot 4H_2O$ nanowire composites were firstly stabilized in air at 150°C for 24 h. The samples were finally pre-annealed in two temperature steps at 280°C for 1 h and at 500 °C for 1 h in air to obtain porous nanowires.

Characterization of the nanowires

Nickel acetate tetrahydrate ($Ni(CH_3COO)_2 \cdot 4H_2O$) was employed as precursor to achieve PVP/($Ni(CH_3COO)_2$ nanowire composite by an electrospinning method (Supplementary information). A non-equilibrium calcination process triggered the thermal decomposition of the PVP/($Ni(CH_3COO)_2$ nanowire composites in air which resulted in NiO nanowires. The average diameter of polyvinylpyrrolidone $Ni(CH_3COO)_2$/PVP composite nanowires is ~ 650 nm (Figure S2a). To achieve the NiO nanowires, an optimized calcination process is introduced to the composite nanowires. Firstly, the composite was stabilized in air at 150℃ for 24 hours, pre-oxidized at 280℃ for 1 hour, then calcined at 500℃ for 1 hour. After the thermal

process, NiO nanowires were obtained as shown in the Figure S2b. The structure of as-synthesized NiO nanowires is identified to be of the cubic symmetry with the space group of Fm$\bar{3}$m (225) by indexed X-ray diffraction patterns (Figure S2c).

Different orientation study in NiO

NiO crystal structure belongs to cubic system. The angle between crystal planes (h_1,k_1,l_1) and (h_2,k_2,l_2) in cubic crystal can be calculated using:

$$cos\,\theta = \frac{h_1h_2 + k_1k_2 + l_1l_2}{\sqrt{(h_1^2 + k_1^2 + l_1^2)(h_2^2 + k_2^2 + l_2^2)}}$$

We calculated an angle of 19° between the (110) and (120) crystal planes, and an angle of 27° between the (110) and (130) crystal planes. The (120) plane is the nearest neighboring plane to the (110) plane , and the plane of (130) is the second nearest neighboring plane.

Facet notation for high Miller index surfaces

To determine the Miller index of a surface, Somorjai et al has demonstrated a notation method for surface index of cubic material.[S1] In fcc lattice, a high Miller index (hkl) of a surface with (h_t k_t l_t) terraces, (h_s k_s l_s) steps, and a step-to-terrace atom ratio of $n_{h_tk_tl_t}^{uc}$: $n_{h_sk_sl_s}^{uc}$ can be written as:

$$(hkl) = a_t(h_tk_tl_t) + a_s(h_sk_sl_s)$$

$$n_{h_tk_tl_t}^{uc}: n_{h_sk_sl_s}^{uc} = P_ta_t: P_sa_s$$

Where a_t, a_s are the vector decomposition coefficients of the vector (h_t k_t l_t) and (h_s k_s l_s), respectively. h_t, k_t, l_t, must be an irreducible set of integers and so is h_s, k_s, l_s. P_t=4 when h_t, k_t, l_t are all odd; P_t=2 when h_t, k_t, l_t are not all odd. P_s=4 when h_s, k_s, l_s are all odd; P_s=2 when h_s, k_s, l_s are not all odd. This method helps us to rapidly find the

Miller index of a curved surface in an fcc lattice.

Computational methods

All the calculations were performed by using Vienna ab initio Simulation package (VASP).[S2] The frozen-core all-electron projector augmented wave (PAW) model with Perdew-Burke-Ernzerhof (PBE) function was employed to describe the interactions between core and electrons. An energy cutoff of 300 eV was used for the plane-wave expansion of the electronic wave function. The force and energy convergence criterion was set to 0.01 eV/Å and 10^{-5} eV, respectively. Only Gamma point was performed for the first Brillouin zone. Atomic models were built by 5 to 9 NiO layers with the thickness of about 5 Angstrom for all of the facets, whose surfaces are exposed to the vacuum in the unit cell for VASP computations (Figure S6).

Electrochemical characterization

All electrochemical experiments were performed in a three-electrode system at room temperature with an electrochemical analyzer (660D CH Instrument, purchased from Shanghai Chenhua Instrument Co., Ltd.). Naked glass carbon electrode (GCE, d = 3 mm) or GCE deposited with NiO (～5 μg) were used as the working electrodes to obtain cyclic voltammograms. Ag/AgCl (3 M KCl) electrode was used as the reference electrode and the Pt wire as the counter electrode. Prior to the tests, GCE was cleaned by polished α-Al_2O_3 powder (1 μm, 0.3 μm and 50 nm, respectively). Then the electrode was cleaned by deionized water, ethanol and dried in air. The catalyst solution was prepared by adding 10% (Volume) nafion into a mixture of NiO ethanol solution and stirred until the jelly formed. The resulting solution was 5 mg/mL

and it was deposited on the surface of GCE (1μL). The final deposited catalyst was 5μg. The different NiO samples were named as NiO (10%), NiO (30%), NiO (50%) for convenience. The naked GCE electrode was used as the control test.

All the cyclic voltammograms were collected at 50 mV/s in a 1.0 M potassium phosphate buffer solution (Pi, pH = 7.0). The CV scans were recorded in a range of -2.0V~0 V versus Ag/AgCl electrode (3 M KCl). There was an iR drop for compensation and no stirring was used for the CV tests. All the potential in this work was referenced versus reversible hydrogen electrode (RHE).

The H_2 was detected by using gas chromatography (SP-6890, nitrogen as a carrier gas) equipped with thermal conductivity detector (TCD). The experiment was carried out in a gas-tight electrochemical cell and the solution was degassed by bubbling with high purity N_2 for 1 h with vigorous stirring. After that, 5 mL of nitrogen was removed from the flask and 5 mL of Methane (760 Torr) was added to the flask to serve as the internal standard for gas chromatography (GC) measurements. After electrolysis for defined periods of time (every other five minutes), a fully deaerated 100 μL gas-tight syringe was used to withdraw a sample of the gas from the reaction vessel headspace. Aliquots (100 μL) of this gas were immediately injected into a SP-6890 gas chromatograph fitted with a thermal conductivity detector for analysis (N_2 as the carrier gas). The GCE deposited with 5μg NiO was used as the working electrode. The reference electrode was fixed at a position less than 1 cm from the surface of the catalyst film. Bulk electrolysis was carried out at -1.5 V (877 mV overpotential vs RHE) in 1.0 M Pi solution for 2 h without iR drop compensation. The

volume of the solution and the volume of the headspace in the working compartment were measured as 40 mL and 46 mL, respectively. The theoretical amount of H_2 was calculated by dividing the passing charge by 2F (F was the faraday constant) and the experimental amount of H_2 was calculated by converting the measured partial pressure of H_2 into mole numbers.

The catalyst stability of the electrochemical activity for the H_2 evolution was measured by chronopotentiometry. The current density was fixed at 10 mA/cm^2. There was no significant change of the overpotential during the catalysis after 24 hours.

Photocatalytic hydrogen production

Different ratios of NiO nanowires (0.5% ~ 3.0%) were added to a TiO_2 suspension in deionized water, and the final mixtures were sonicated for 30 min. Photocatalytic activities for hydrogen production were carried out in a 50 mL round-bottom flask containing 20 mL of deionized water, 10 mg TiO_2-NiO, 1 mL TEOA, 0.5 mM Eosin-Y dye. Sacrificial electron donor TEOA and photosensitizers Eosin-Y dye play important roles for the efficient photocatalytic reactions. In the presence of TEOA, it can promote the transition of fluorescent dye from singlet to triplet state, then the life of triplet state becomes longer than that of the singlet. So it is more favorable to generate the excited states of EY^{2-} (Eosin-Y), which enhances the utilization efficiency of excited state EY^{2-} species.[S3-S5] The container was capped with a rubber stopper and extensively deaerated by bubbling N_2 gas through the solution for over 20

minutes. After that, 5 mL of Methane (760 Torr) were added to the flask to serve as the internal standard for gas chromatography (GC) measurements. The photocatalytic reactions were carried out in a photoreactor equipped with 300W Xe lamp under visible light irradiation (λ > 420 nm, Perfect Light, PLS-SXE300, China). After light irradiation for a certain time, syringe was used to take a sample of the gas from the headspace of the reaction vessel. The gas was immediately injected into a SP-6890 gas chromatograph fitted with a thermal conductivity detector for analysis (N_2 as the carrier gas). Three cycles for the H_2 generation rate experiments (Figure 4c) were carried out. After each cycle, the reaction system was evacuated. Under the dark environment, no other gaseous components were detected.

The dye's influence on the catalytic properties of NiO can be attributed to the heavy-atom effect of Br substituents, which can promote the efficient formation of long-lived triplet states for EY^{2-} from its photoexcited singlet state.[S4] Too much dye could block the active sites in NiO to contact the reactants.

Powder X-ray diffractometer (XRD)

The crystal phase and phase composition of the NiO samples were determined by powder X-ray diffraction (XRD, D/max-TTR III) using graphite monochromatized Cu Ka radiation of 1.54178 Å, operating at 40 kV and 200 mA. The scanning rate was 10° min^{-1} from 20˚ to 70˚ in 2θ.

References

S1. Van Hove, M. A. & Somorjai, G. A. A new microfacet notation for high-Miller-index surfaces of cubic materials with terrace, step and kink structures.

Surf. Sci. **92**, 489-518 (1980).

S2. Kresse, G. & Furthmüller, J. Efficient iterative schemes for ab initio total-energy calculations using a plane-wave basis set. *Phys. Rev. B* **54**, 11169-11186 (1996).

S3. Lazarides, T., McCormick, T., Du, P., Luo, G., Lindley, B. & Eisenberg, R. Making hydrogen from water using a homogeneous system without noble metals. *J. Am. Chem. Soc.* **131**, 9192-9194 (2009).

S4. Shimidzu, T., Iyoda, T. & Koide, Y. An advanced visible-light-induced water reduction with dye-sensitized semiconductor powder catalyst. *J. Am. Chem. Soc.* **107**, 35-41 (1985).

S5. Min, S. & Lu, G. Sites for high efficient photocatalytic hydrogen evolution on a limited-layered MoS2 cocatalyst confined on graphene sheets—the role of graphene. *J. Phys. Chem. C.* **116**, 25415-25424 (2012).

(a)

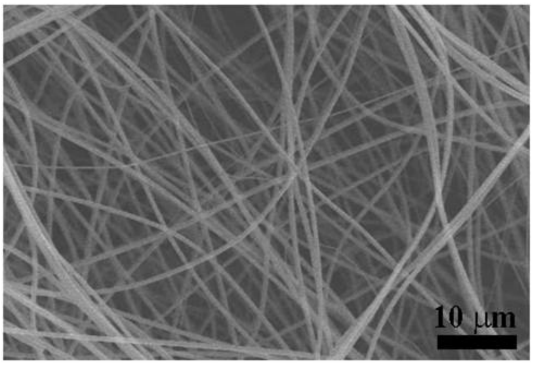

(b)

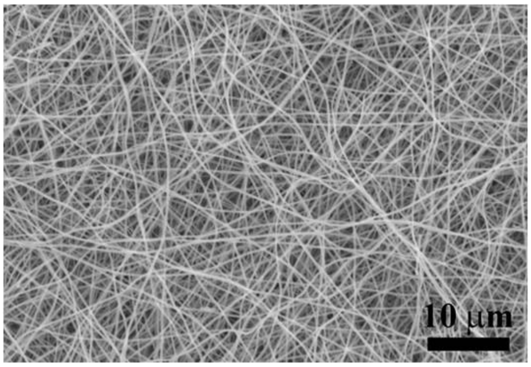

(c)

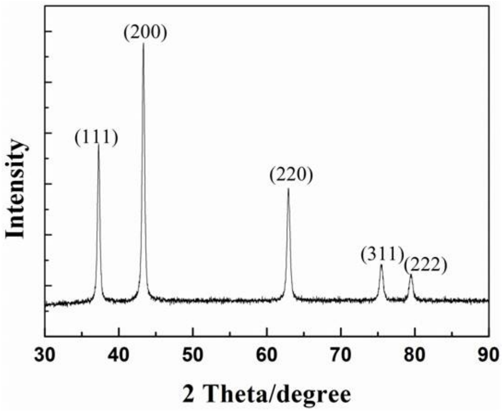

Figure S1. (a) SEM image of the $Ni(CH_3COO)_2$/PVP composite nanowires. (b) SEM image of the porous NiO nanowires. (c) XRD pattern of the as-synthesized NiO nanowires.

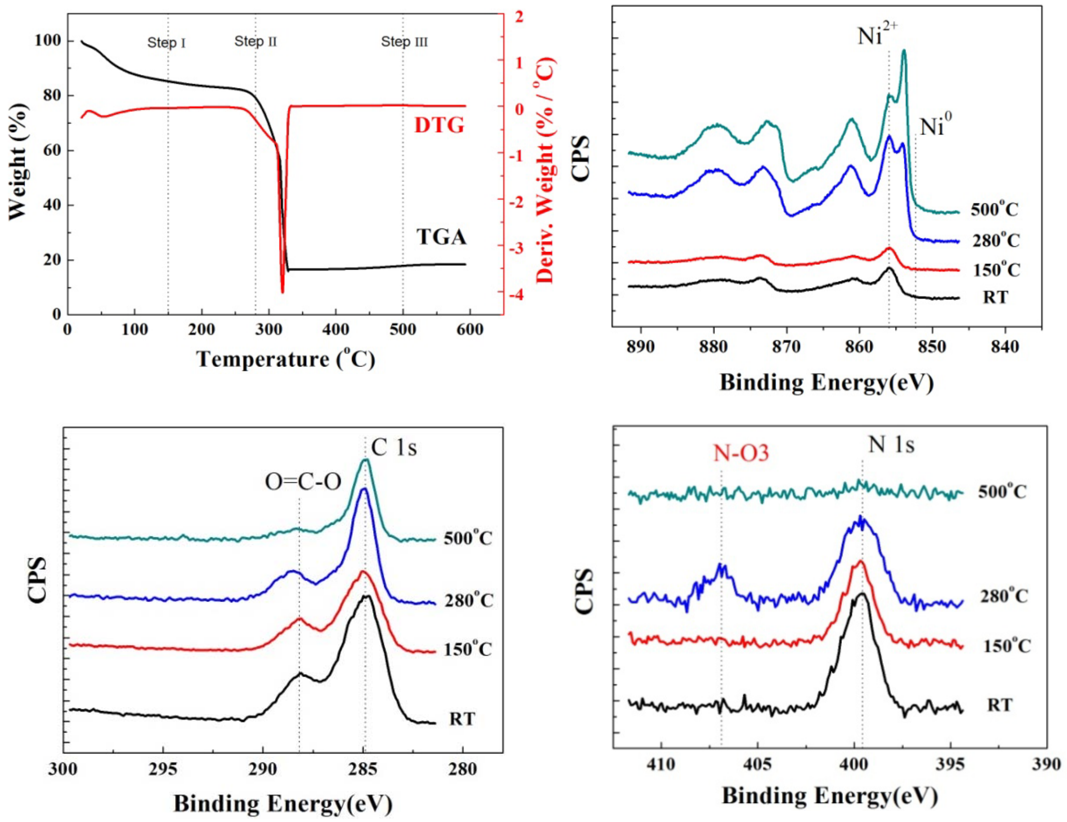

Figure S2. (a) TGA (black color) and DTG (red color) curves of the $Ni(CH_3COO)_2$/PVP composite nanowires measured at a heating rate of 10℃/min in air. The porous NiO nanowires are formed at ~ 325 °C. Step I stabilization stage, step II pre-oxidation stage, step III calcination process. (b-d) XPS spectra collected during the thermal decomposition of the $Ni(CH_3COO)_2$/PVP composite nanowires. The peak at 284.9 eV observed in the sample at 500℃ was due to the contamination of the adventitious carbon during the XPS measurements.

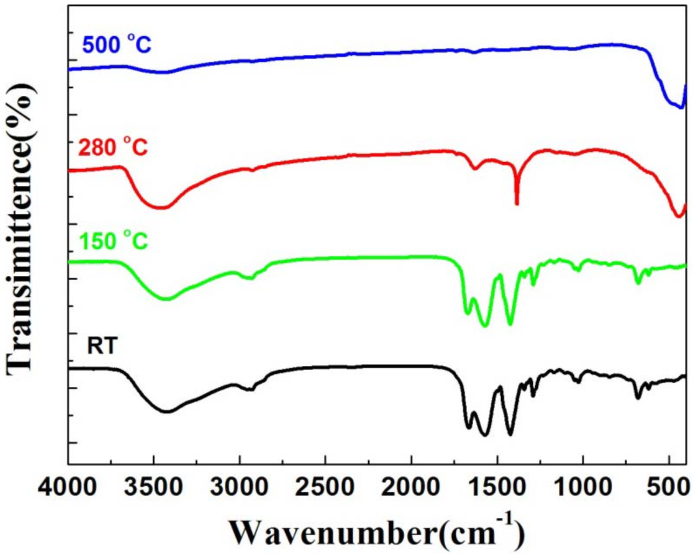

Figure S3. FTIR spectra of (a) As electrospun $Ni(CH_3COO)_2$/PVP nanowires (b) Stabilized nanowires (c) Pre-oxidized nanowires (d) Calcinated NiO nanowires

Table S1: Synthesis process for NiO nanowires

Sample Code	Electrospinning Step		Calcination steps				
	Weight of $NiAc_2 \cdot 4H_2O$	Volume of DMF	Step 1		Step 2		Heating Rate
			Temperature	Time	Temperature	Time	
NiO_10%	1g	10ml	280℃	1h	500℃	1h	2℃/min
NiO_30%	3g						
NiO_50%	5g						

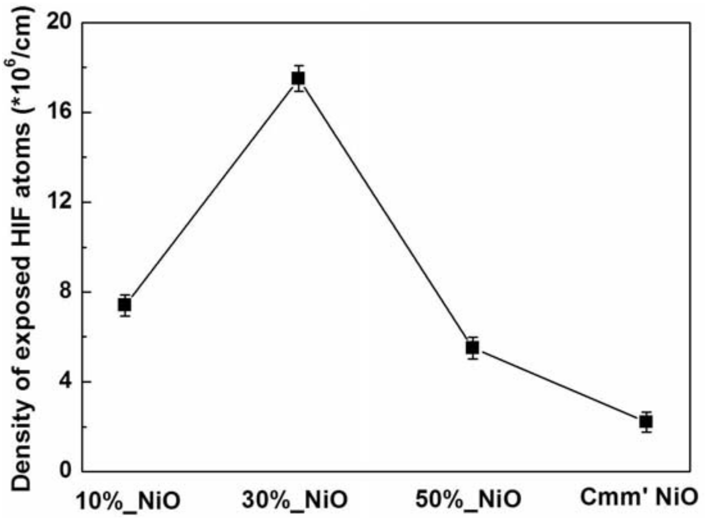

Figure S4. The linear density of exposed HIF atoms in NiO nanoparticles with a zone axis along [110] direction.

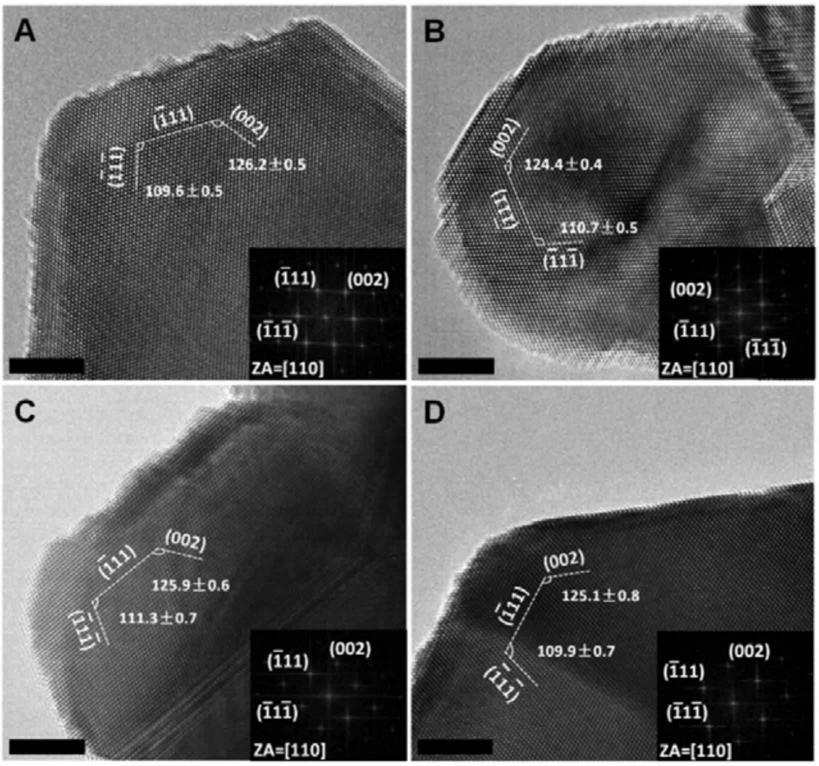

Figure S5. Representative HRTEM images of a) NiO_10%, b) NiO_30%, c) NiO_50% and d) commercial NiO. The insets are the corresponding FFT patterns of HRTEM images. The zone axis is along [110]. The labeled measured interfacial angles are in good agreement with the calculated values. Scale bar is 5 nm.

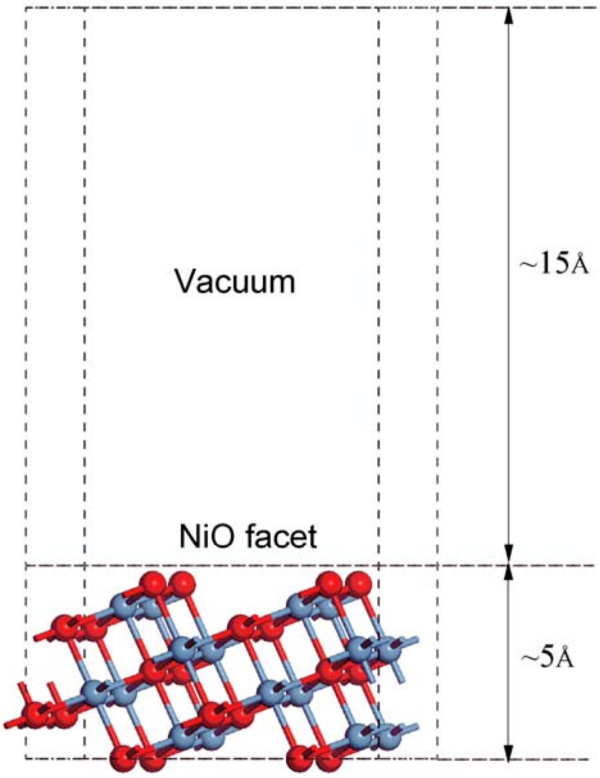

Figure S6. The atomic model of NiO used for electronic and geometric structure simulations.

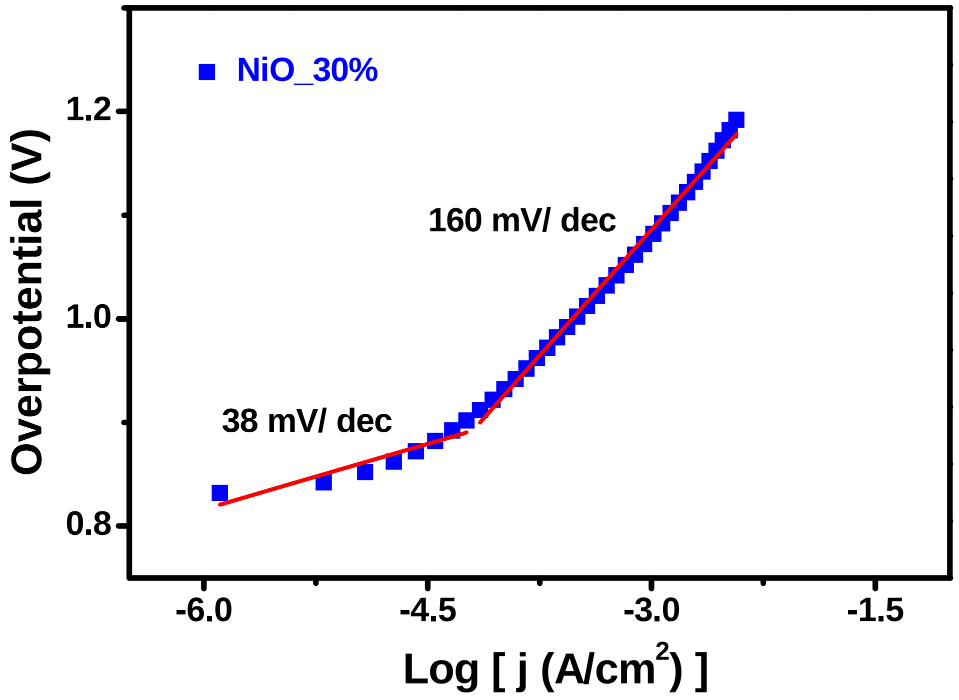

Figure S7. The Tafel plot demonstrating the electrocatalytic activity of NiO_30% in an electrochemical catalytic hydrogen production. The linear approximation slopes of the curve are labeled in the plot.

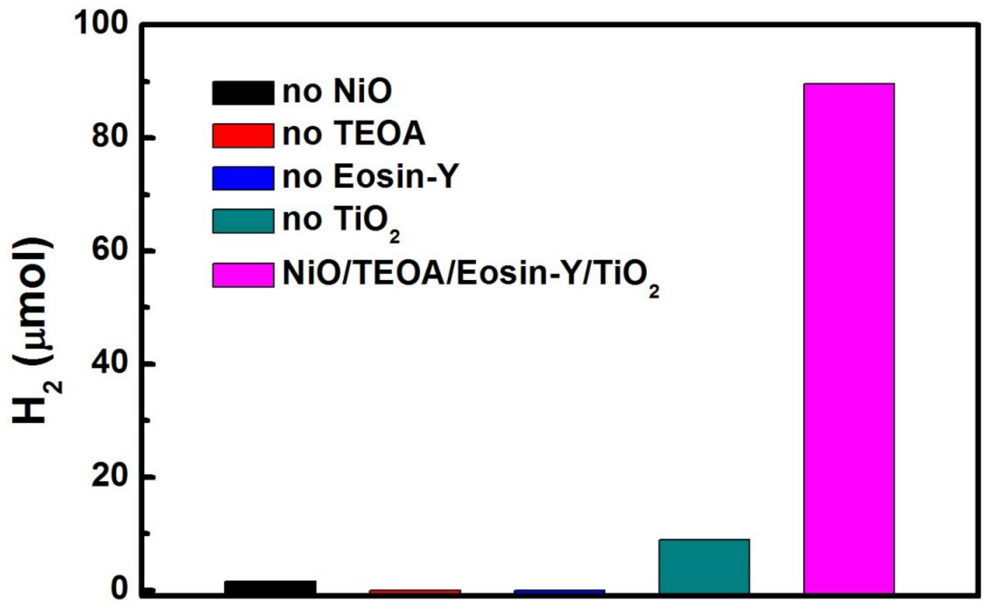

Figure S8. The evolution of hydrogen production with different composition of reactants in photocatalytic HER. In the presence of as-synthesized NiO as co-catalyst in HERs, the hydrogen production exhibits a substantial increase.

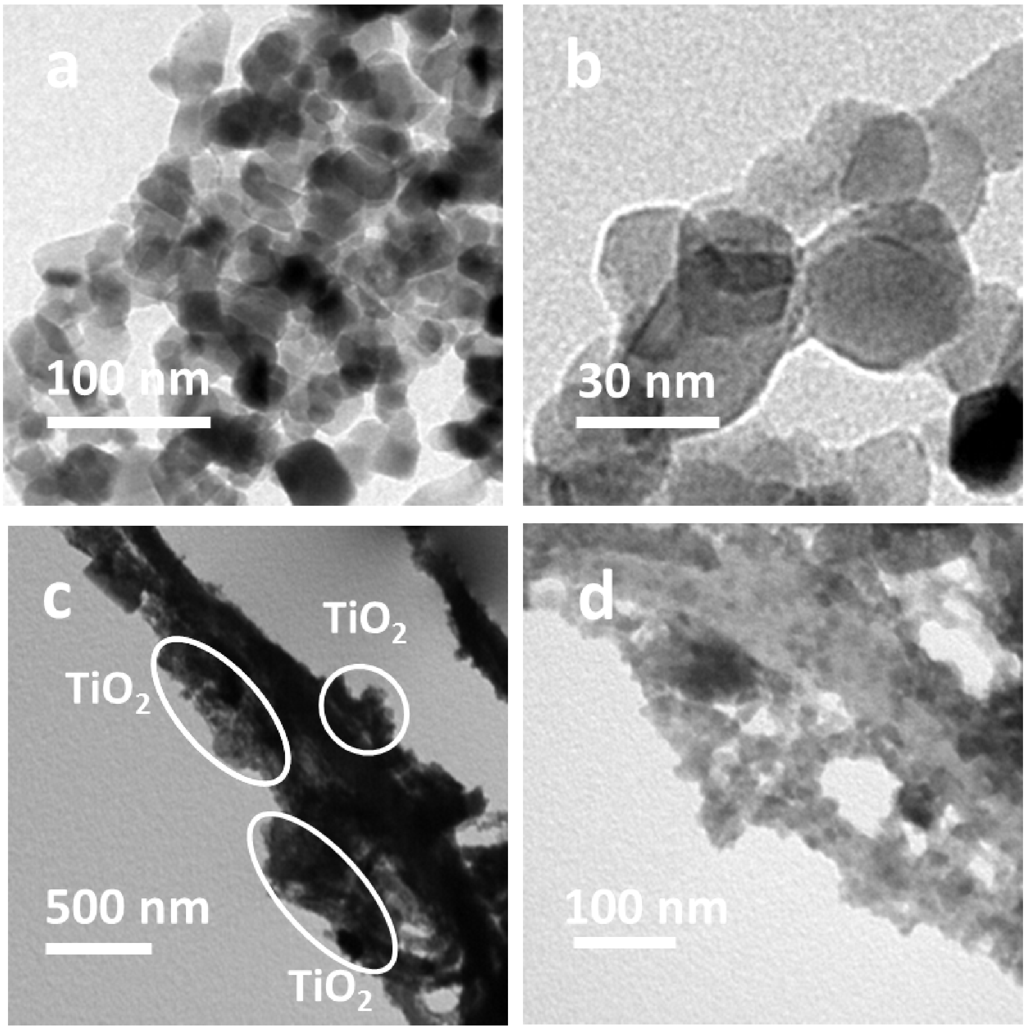

Figure S9. The TEM images of **(**a) and (b) TiO2 nanoparticles. The TEM images of (c) and (d) TiO2/NiO photcatalysts.

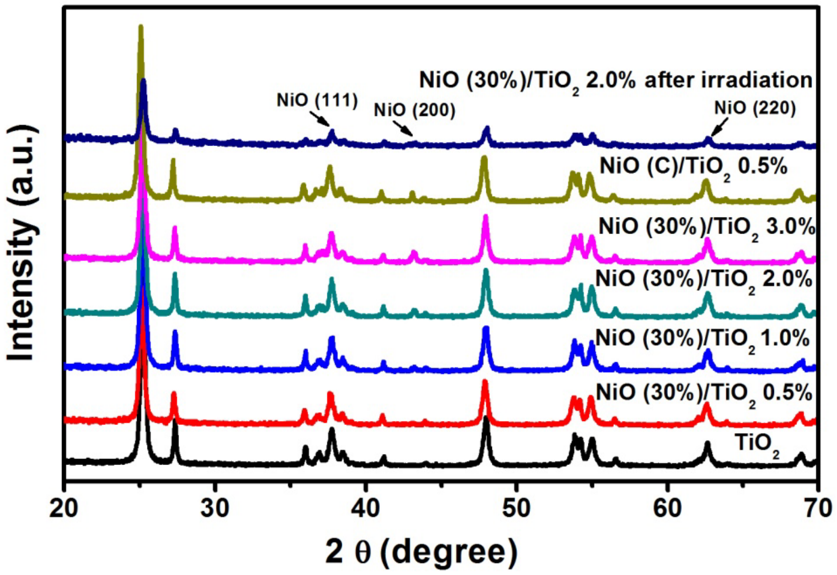

Figure S10. XRD patterns of NiO/TiO_2 photocatalysts.

The specific surface area of NiO samples was measured by Quantachrome Instrument Autosorb-iQ. The NiO nanowires were loaded into the chamber, then pretreated at 300℃ for 12h under vacuum atmosphere. After that, the Brunauer-Emmett-Teller (BET) measurements were carried out at 77K under N_2 atmosphere. Finally, the measured specific surface area were obtained as listed in the Table S2.

Table S2: The BET measurements of NiO nanowires

Sample name	10%	30%	50%
Specific surface area (m^2/g)	29.295	16.624	24.247
